# Kinetics and Quantitative Structure—Activity Relationship Study on the Degradation Reaction from Perfluorooctanoic Acid to Trifluoroacetic Acid

**DOI:** 10.3390/ijms150814153

**Published:** 2014-08-14

**Authors:** Chen Gong, Xiaomin Sun, Chenxi Zhang, Xue Zhang, Junfeng Niu

**Affiliations:** 1 Environment Research Institute, Shandong University, Jinan 250100, China; E-Mails: gongchen06@lzu.edu.cn (C.G.); sdzhangcx@163.com (C.Z.); zxue_117@126.com (X.Z.); 2 State Key Laboratory of Water Environment Simulation, School of Environment, Beijing Normal University, Beijing 100875, China

**Keywords:** electrochemical degradation, PFOA, rate constants, quantitative structure-activity relationship

## Abstract

Investigation of the degradation kinetics of perfluorooctanoic acid (PFOA) has been carried out to calculate rate constants of the main elementary reactions using the multichannel Rice-Ramsperger-Kassel-Marcus theory and canonical variational transition state theory with small-curvature tunneling correction over a temperature range of 200~500 K. The Arrhenius equations of rate constants of elementary reactions are fitted. The decarboxylation is role step in the degradation mechanism of PFOA. For the perfluorinated carboxylic acids from perfluorooctanoic acid to trifluoroacetic acid, the quantitative structure–activity relationship of the decarboxylation was analyzed with the genetic function approximation method and the structure–activity model was constructed. The main parameters governing rate constants of the decarboxylation reaction from the eight-carbon chain to the two-carbon chain were obtained. As the structure–activity model shows, the bond length and energy of C1–C2 (*R*_C1–C2_ and *E*_C1–C2_) are positively correlated to rate constants, while the volume (V), the energy difference between *E*_HOMO_ and *E*_LUMO_ (Δ*E*), and the net atomic charges on atom C_2_ (*Q*_C2_) are negatively correlated.

## 1. Introduction

Perfluorinated carboxylic acids (PFCAs) have been widely used in industry as surfactants, surface treatment agents, metal coating, fire retardants and carpet cleaners for many years [1,2,3,4]. As a typical perfluorinated acid, the production of perfluorooctanoic acid (PFOA) worldwide exceeded 1000 metric tons in 2004 [5]. PFOA is a new persistent organic pollutant and difficult to decompose in the environment [6]. Due to its long-range oceanic and atmospheric transportation ability, PFOA has been detected in various environmental matrices such as water, dust, sediments and living tissues [7,8,9]. Research shows that levels of PFOA in wildlife range from 0.05 ng/mL in the blood of cod collected from European waters to 8.14 ng/mL in the plasma of loggerhead sea turtles from North America [10,11]. It can be also accumulated in creatures through water, food and atmosphere, causing the decrease in fertility rate, birth weight and other immune system diseases [12,13,14,15,16,17,18]. Although the environmental protection agency of the United States (US-EPA) and the European environment agency (EEA) have adopted an industrial program in order to reduce the global emissions of PFOA [19,20], the remained PFOA in the environment still has potential risk on humans. So, it is important and urgent to find an effective degradation method.

Recently, electrochemical treatment of PFOA has been considered one of the most promising methods due to its strong oxidation and environmental compatibility [6]. What is more, Hoffman *et al.* found that PFOA-contaminated drinking water is a significant contributor to PFOA levels in serum [21]. Researchers have found that PFOA could be degraded over boron-doped diamond (BDD) film electrode and Ti/SnO_2_-Sb-Bi electrode [22,23,24]. The reaction mechanism is proposed as the direct electrochemical oxidation cleaves the C-C bond between the C_7_F_15_ and COOH in PFOA to generate a C_7_F_15_ radical and CO_2_ firstly [24]. Zhuo *et al.* studied the electrochemical oxidation of PFOA using Ti/SnO_2_-Sb-Bi anode, and they found that after 2 h electrolysis, over 99% of PFOA was degraded with a first-order kinetic constant of 1.93 h^−1^. Then, the degradation mechanism was revealed according to the intermediate products detected [23]. Lin *et al.* studied the electrochemical degradation efficiency of PFOA using different anode, current density, pH value, plate distance and concentration. They also put forward a reaction mechanism in accordance with experiment results [6]. Although much work has been done to study the degradation effect of electrochemistry, the degradation mechanism has been partly revealed, and the rate constants of elementary reactions have not been reported so far. The reaction rate is helpful to find an optimal reaction way, then a kinetics study is needed. Furthermore, the quantitative structure–activity relationship (QSAR) analysis is helpful to understand the reaction mechanism.

In this study, the geometrical parameters are optimized at the MPWB1K/6-31 + G(d, p) level. On the basis of the quantum chemical information, the rate constants are calculated using the multichannel Rice-Ramsperger-Kassel-Marcus (RRKM) theory and canonical variational transition state theory (CVT) with small-curvature tunneling (SCT) correction over a wide temperature range of 200-500 K. Then, the kinetics study has been performed to calculate the rate constants of elementary reactions, and the quantitative structure–activity relationship from perfluorooctanoic acid to trifluoroacetic acid with rate constants is analyzed in order to explore the main factors affecting the rate constant of decarboxylation reaction.

## 2. Results and Discussion

**Figure 1:**
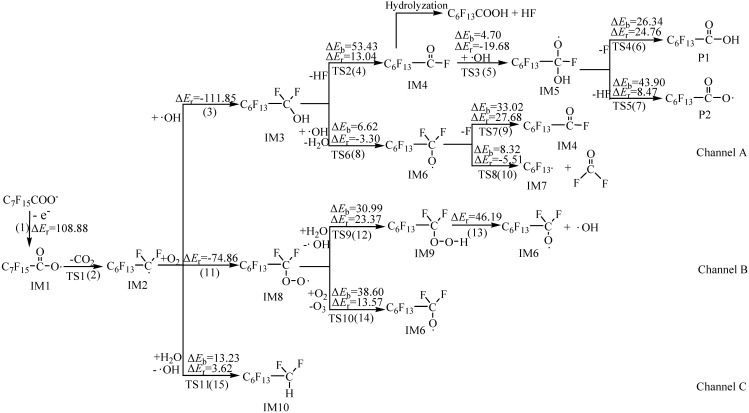
The main possible reaction paths of electrochemical degradation of PFOA embedded with the potential barriers Δ*E*_b_ (kcal·mol^−1^) and reaction heats Δ*E*_r_ (kcal·mol^−1^).

**Figure 2:**
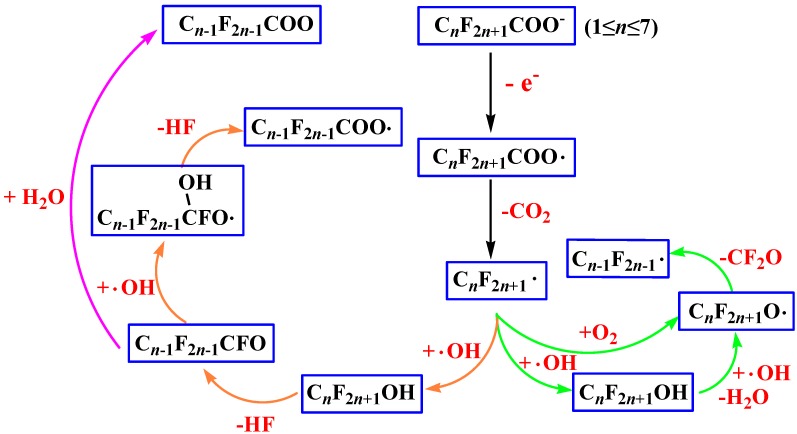
The three reaction circles in the electrochemical degradation pathways.

The main possible reaction paths of electrochemical degradation of PFOA are drawn in Figure 1. Figure 2 shows two reaction circles in the electrochemical degradation pathways. The chemical structures of transition states in electrochemical degradation reactions are shown in Figure S1.

### 2.1. Reaction Mechanism

The detailed electrochemical mineralization mechanism of PFOA has been analyzed in previous study [25]. The first step of electrochemical degradation is the electron transfer process from carboxylic acid to anode in which the intermediate IM1 is generated [24]. Then, perfluoroheptyl radical IM2 and CO_2_ are produced from IM1 via the transition state TS1, which is a decarboxylation reaction. In the subsequent reactions, IM2 may continue to react with OH, O_2_ and H_2_O in the electrolytic cell. Channel A shows the reactions initiated by OH. The addition reaction of IM2 with OH generates adduct IM3, which is a barrierless process. Perfluoroheptanoyl fluoride IM4 is formed through a HF desorption process in the four-membered ring of IM3 via the transition state TS2. Obviously, IM4 can be hydrolyzed to perfluoroheptanoic acid (PFHA) P1 and hydrofluoric acid HF. What is more, IM4 also can continue to react with OH to generate adduct IM5 via the transition state TS3. Then, the fluorine atom will be removed to form P1 via the transition state TS4. A HF desorption process in the four-membered ring of IM5 can also occur to generate PFHA radical P2 via the transition state TS5. Obviously, the HF desorption process of IM3 is challenged due to its high potential barrier of 53.43 kcal·mol^−1^. An easier way lies in the hydrogen atom of the IM3 being abstracted by OH to form intermediate IM6 via the transition state TS6, and the potential barrier is 6.62 kcal·mol^−1^. Then, IM6 can remove a fluorine atom to produce the IM4 via the transition state TS7, or be decomposed into perfluorohexyl radical IM7 and difluorophosgene via the transition state TS8. The above reaction barriers are 33.02 and 8.32 kcal·mol^−1^, respectively. Difluorophosgene can be hydrolyzed to the final products HF and CO_2_. Obviously, the latter path occurs much more easily thermodynamically.

Channel B depicts the reactions initiated by oxygen. IM2 reacts with O_2_ to generate adduct IM8, which is a barrierless reaction. The oxygen atom of IM8 can be abstracted by OH and O_2_ to form IM9 and IM6 via the transition state TS9 and TS10, respectively. IM9 can also be decomposed into IM6 and OH, but considering the high potential barrier of TS9, it is a difficult reaction.

The reaction of IM2 and H_2_O is shown in Channel C. The hydrogen atom of H_2_O is abstracted by IM2 and IM10 is generated via the transition state TS11. The potential barrier and endothermic energy are 13.23 and 3.62 kcal·mol^−1^, respectively.

As shown in Figure 2, the electrochemical degradation of PFOA has three circles. The first one is the degradation from PFOA radical to PFHpA radical, the subsequent degradation process is from PFHpA to PFHxA radical, until the perfluorinated acetic acid finally decomposes into CO_2_ and HF. The second one is the degradation from PFOA to PFHpA, and the final products are same as for the first one. The last one is the degradation from C_7_F_15_ radical to C_6_F_13_ radical, and C_6_F_13_ radical to C_5_F_11_ radical, until it decomposes to CF_3_ radical, which represents complete degradation.

### 2.2. Rate Constants

The RRKM, TST and the CVT with SCT correction method are used to calculate the rate constants. The RRKM method is employed to calculate the rate constants of elementary reactions without barriers, such as elementary reactions (3), (11), and (13). Except for the above elementary reactions of PFOA, the rate constants of decarboxylation reactions of PFCAs radicals (~C8–C2) are also calculated for PFOA when it is degraded successfully after decarboxylation reaction in experiments [25,26]. The rate constants of elementary reactions (2)–(15) and decarboxylation reactions (~C8–C2) at the temperature range of 200–500 K are reported and listed in Table 1 and Table 2, respectively. The reaction pressure is adopted at atmospheric pressure.

**Table 1:** The rate constants of decarboxylation reactions (~C_8_–C_2_) at the temperature range of 200–500 K (CVT/SCT).

*T* (K)	*k*_8C-7C_^a^	*k*_7C-6C_^a^	*k*_6C-5C_^a^	*k*_5C-4C_^a^	*k*_4C-3C_^a^	*k*_3C-2C_^a^	*k*_2C-1C_^a^
200	1.13 × 10^12^	4.21 × 10^10^	1.34 × 10^11^	2.05 × 10^11^	3.81 × 10^11^	5.17 × 10^11^	7.62 × 10^11^
220	1.26 × 10^12^	5.01 × 10^10^	1.54 × 10^11^	2.50 × 10^11^	4.25 × 10^11^	5.22 × 10^11^	7.58 × 10^11^
240	1.39 × 10^12^	5.80 × 10^10^	1.74 × 10^11^	2.95 × 10^11^	4.67 × 10^11^	5.28 × 10^11^	7.55 × 10^11^
260	1.52 × 10^12^	6.57 × 10^10^	1.92 × 10^11^	3.40 × 10^11^	5.07 × 10^11^	5.33 × 10^11^	7.17 × 10^11^
280	1.64 × 10^12^	7.32 × 10^10^	2.10 × 10^11^	3.85 × 10^11^	5.45 × 10^11^	5.38 × 10^11^	7.19 × 10^11^
298.15	1.75 × 10^12^	7.97 × 10^10^	2.25 × 10^11^	4.25 × 10^11^	5.79 × 10^11^	5.42 × 10^11^	7.21 × 10^11^
320	1.87 × 10^12^	8.72 × 10^10^	2.43 × 10^11^	4.73 × 10^11^	6.17 × 10^11^	5.48 × 10^11^	7.25 × 10^11^
340	1.97 × 10^12^	9.38 × 10^10^	2.58 × 10^11^	5.16 × 10^11^	6.50 × 10^11^	5.52 × 10^11^	7.29 × 10^11^
360	2.08 × 10^12^	1.00 × 10^11^	2.73 × 10^11^	5.58 × 10^11^	6.82 × 10^11^	5.56 × 10^11^	7.33 × 10^11^
380	2.17 × 10^12^	1.06 × 10^11^	2.86 × 10^11^	5.98 × 10^11^	7.13 × 10^11^	5.60 × 10^11^	7.37 × 10^11^
400	2.27 × 10^12^	1.12 × 10^11^	2.99 × 10^11^	6.38 × 10^11^	7.42 × 10^11^	5.64 × 10^11^	7.41 × 10^11^
450	2.48 × 10^12^	1.25 × 10^11^	3.29 × 10^11^	7.32 × 10^11^	3.19 × 10^11^	5.73 × 10^11^	7.52 × 10^11^
500	2.68 × 10^12^	1.36 × 10^11^	3.55 × 10^11^	8.19 × 10^11^	3.38 × 10^11^	5.82 × 10^11^	7.82 × 10^11^

^a^ The unit of the rate constant is s^−1^.

**Table 2:** The rate constants of elementary reactions (2)–(15) at the temperature range of 200–500 K (CVT/SCT).

*T* (K)	*k*(2) ^a^	*k*(3) ^b^	*k*(4) ^a^	*k*(5) ^b^	*k*(6) ^a^	*k*(7) ^a^	*k*(8) ^b^	*k*(9) ^a^	*k*(10) ^a^	*k*(11) ^b^	*k*(12) ^b^	*k*(13) ^a^	*k*(14) ^b^	*k*(15) ^b^
200	1.13 × 10^12^	3.21 × 10^−12^	2.80 × 10^−42^	1.06 × 10^−22^	1.64 × 10^−20^	1.54 × 10^−32^	1.15 × 10^−24^	4.21 × 10^−24^	9.70 × 10^6^	9.92 × 10^−18^	1.21 × 10^−58^	4.75 × 10^−21^	2.60 × 10^−70^	3.73 × 10^−38^
220	1.26 × 10^12^	4.33 × 10^−12^	1.98 × 10^−37^	3.71 × 10^−22^	4.83 × 10^−18^	1.18 × 10^−28^	6.70 × 10^−24^	7.50 × 10^−21^	2.99 × 10^7^	1.75 × 10^−17^	3.50 × 10^−56^	1.91 × 10^−20^	1.80 × 10^−65^	3.63 × 10^−36^
240	1.39 × 10^12^	5.58 × 10^−12^	2.18 × 10^−33^	1.06 × 10^−21^	1.12 × 10^−15^	2.04 × 10^−25^	2.96 × 10^−23^	3.86 × 10^−18^	7.70 × 10^7^	2.82 × 10^−17^	3.85 × 10^−54^	6.17 × 10^−20^	1.98 × 10^−61^	1.67 × 10^−34^
260	1.52 × 10^12^	6.93 × 10^−12^	5.74 × 10^−30^	2.59 × 10^−21^	1.13 × 10^−13^	1.12 × 10^−22^	1.05 × 10^−22^	7.59 × 10^−16^	1.72 × 10^8^	4.28 × 10^−17^	2.03 × 10^−52^	1.69 × 10^−19^	5.27 × 10^−58^	4.33 × 10^−33^
280	1.64 × 10^12^	8.35 × 10^−12^	4.90 × 10^−27^	5.62 × 10^−21^	5.89 × 10^−12^	2.49 × 10^−20^	3.14 × 10^−22^	7.02 × 10^−14^	3.46 × 10^8^	6.18 × 10^−17^	6.00 × 10^−51^	4.07 × 10^−19^	4.59 × 10^−55^	7.15 × 10^−32^
298.15	1.75 × 10^12^	9.69 × 10^−12^	1.02 × 10^−24^	1.04 × 10^−20^	1.35 × 10^−10^	1.79 × 10^−18^	7.52 × 10^−22^	2.52 × 10^−12^	6.01 × 10^8^	8.28 × 10^−17^	8.70 × 10^−50^	8.24 × 10^−19^	9.84 × 10^−53^	6.64 × 10^−31^
320	1.87 × 10^12^	1.13 × 10^−11^	2.84 × 10^−22^	2.01 × 10^−20^	3.63 × 10^−9^	1.62 × 10^−16^	1.91 × 10^−21^	1.09 × 10^−10^	1.08 × 10^9^	1.14 × 10^−16^	1.45 × 10^−48^	1.75 × 10^−18^	2.84 × 10^−50^	7.02 × 10^−30^
340	1.97 × 10^12^	1.29 × 10^−11^	5.84 × 10^−20^	3.44 × 10^−20^	5.12 × 10^−8^	6.01 × 10^−15^	4.05 × 10^−21^	2.24 × 10^−9^	1.73 × 10^9^	1.48 × 10^−16^	1.37 × 10^−47^	3.25 × 10^−18^	2.70 × 10^−48^	4.71 × 10^−29^
360	2.08 × 10^12^	1.44 × 10^−11^	3.25 × 10^−18^	5.56 × 10^−20^	5.37 × 10^−7^	1.49 × 10^−13^	7.95 × 10^−21^	3.29 × 10^−8^	2.65 × 10^9^	1.87 × 10^−16^	1.01 × 10^−46^	5.66 × 10^−18^	1.56 × 10^−46^	2.58 × 10^−28^
380	2.17 × 10^12^	1.60 × 10^−11^	1.18 × 10^−16^	8.60 × 10^−20^	4.40 × 10^−6^	2.64 × 10^−12^	1.46 × 10^−20^	3.62 × 10^−7^	3.87 × 10^9^	2.32 × 10^−16^	6.05 × 10^−46^	9.40 × 10^−18^	5.93 × 10^−45^	1.19 × 10^−27^
400	2.27 × 10^12^	1.75 × 10^−11^	3.01 × 10^−15^	1.06 × 10^−19^	2.93 × 10^−5^	3.50 × 10^−11^	2.55 × 10^−20^	3.14 × 10^−6^	5.45 × 10^9^	2.84 × 10^−16^	3.01 × 10^−45^	1.49 × 10^−17^	1.57 × 10^−43^	4.77 × 10^−27^
450	2.48 × 10^12^	2.05 × 10^−11^	2.79 × 10^−12^	2.44 × 10^−19^	1.59 × 10^−3^	8.21 × 10^−9^	8.39 × 10^−20^	2.95 × 10^−4^	1.13 × 10^10^	4.42 × 10^−16^	8.93 × 10^−44^	4.08 × 10^−17^	1.64 × 10^−40^	9.11 × 10^−26^
500	2.68 × 10^12^	2.30 × 10^−11^	6.58 × 10^−10^	4.89 × 10^−19^	3.87 × 10^−2^	6.45 × 10^−7^	2.23 × 10^−19^	1.15 × 10^−2^	2.03 × 10^10^	6.42 × 10^−16^	1.35 × 10^−42^	9.42 × 10^−17^	4.37 × 10^−38^	1.00 × 10^−24^

^a^ The unit of the rate constant is s^−1^; ^b^ The unit of the rate constant is cm^3^·molecule^−1^·s^−1^.

For the purpose of comparison, the elementary reaction (2) is taken as an example, and the TST rate constants, the CVT rate constants with the ZCT and the SCT correction are listed in Table S1. The TST rate constant at 200 K is 3.10 × 10^14^ s^−l^, which is 284 times that of the CVT rate constant at the same temperature, 1.09 × 10^12^ s^−l^. It suggests that the variational effect is significant in this reaction, and the higher the temperature is, the weaker the variational effect is. What is more, the tunneling effects are also taken into account to compute the rate constants. It is clear that the CVT constants have no significant difference with the CVT/ZCT and CVT/SCT ones over the temperature range of 200–500 K. It can be seen that the tunneling effect plays a less important role in this rate constant calculation. According to the previous study, the CVT/SCT rate constants are in good agreement with experimental values in a large temperature range [27,28,29]. Then, the results of the CVT/SCT method at 298.15 K are chosen for discussion in this paper. The TST, CVT with the ZCT or the SCT correction rate constants of the main elementary reactions (3)–(15) are listed in Tables S2–S11. Due to the absence of the available experimental values, it is difficult to make a direct comparison of the calculated CVT/SCT rate constants with the experimental values for all the elementary reactions. We hope that our CVT/SCT calculations may provide a good estimation.

The rate constant of the decarboxylation reaction (2) is 1.75 × 10^12^ s^−l^. Obviously, the decarboxylation reaction occurs quite easily after the electron transfer process due to the low potential barrier. The rate constants of elementary reactions (3), (11) and (15) are 9.69 × 10^−12^, 8.28 × 10^−17^ and 6.64 × 10^−31^ cm^3^·molecule^−1^·s^−1^, respectively, which means that Channel A is the main reaction pathway since the rate constant is much higher than those of the other two pathways. As Figure 1 shows, the subsequent reactions of the IM3 in Channel A are divided into two paths, (4) and (8), the rate constant of reaction (4) is 1.02 × 10^−24^ s^−1^, while the rate constant of reaction (8) is 7.52 × 10^−22^ cm^3^·molecule^−1^·s^−1^. The latter is 737 times higher than that of the reaction (4), so the IM3 is easier to react with OH to form the IM6. Then, reaction (10) occurs readily with the rate constant of 6.01 × 10^8^ s^−1^, which demonstrates that the circle of C*_n_*F_2*n*+1_ → C*_n_*_−1_F_2*n*−1_ is the optimal reaction pathway in the degradation process due to the high rate constants. The circle of C*_n_*F_2*n*+1_COO → C*_n_*_−1_F_2*n*−1_COO is achieved by the reaction (5) and (7), and the rate constants are 1.04 × 10^−20^ cm^3^·molecule^−1^·s^−1^ and 1.79 × 10^−18^ s^−1^, respectively. In Channel B, the rate constants of the abstraction reactions (12) and (14) are 8.70 × 10^−50^ and 9.84 × 10^−53^ cm^3^·molecule^−1^·s^−1^, which are difficult to achieve because the reaction barrier is high.

For the rate constants over the temperature range of 200–500 K, the Arrhenius equations, *i.e.*, *k*(*T*) = *A* exp(−*E*a/*RT*), are shown in Table 3. The pre-exponential factor and the activation energy can be obtained from Arrhenius equations. The correlative coefficient *R*^2^ is above 0.997.

**Table 3:** The Arrhenius equations for the rate constants *k*(2)–*k*(15) over the temperature range of 200–500 K.

Reaction	A	*E*a (kJ/mol)	Arrhenius Equation	*R* ^2^
(2)	4.66 × 10^12^	2398.76	*k* *=* 4.66 × 10^12^ exp(−288.52/*T*)	0.997
(3)	9.13 × 10^−11^	5564.81	*k* *=* 9.13 × 10^−11^ exp(−669.33/*T*)	0.999
(4)	3.22 × 10^12^	207,450.90	*k* *=* 3.22 × 10^12^ exp(−24,952/*T*)	0.999
(5)	1.30 × 10^−16^	23,352.36	*k* *=* 1.30 × 10^−16^ exp(−2808.80/*T*)	0.999
(6)	8.05 × 10^10^	118,399.70	*k* *=* 8.05 × 10^10^ exp(−14,241/*T*)	0.999
(7)	7.89 × 10^10^	163,511.40	*k* *=* 7.89 × 10^10^ exp(−19,667/*T*)	0.999
(8)	6.35 × 10^−16^	33,661.72	*k* *=* 6.35 × 10^−16^ exp(−4048.80/*T*)	0.999
(9)	2.30 × 10^12^	136,798.60	*k* *=* 2.30 × 10^12^ exp(−16,454/*T*)	0.999
(10)	3.18 × 10^12^	21,194.88	*k* *=* 3.18 × 10^12^ exp(−2549.30/*T*)	0.999
(11)	8.98 × 10^−15^	11,478.31	*k* *=* 8.98 × 10^−15^ exp(−1380.6/*T*)	0.998
(12)	7.04 × 10^−32^	102,328.70	*k* *=* 7.04 × 10^−32^ exp(−12,308/*T*)	0.998
(13)	5.49 × 10^−14^	27,305.67	*k* *=* 5.49 × 10^−14^ exp(−3284.3/*T*)	0.999
(14)	1.09 × 10^−16^	205,547.02	*k* *=* 1.09 × 10^−16^ exp(−24,723/*T*)	0.999
(15)	7.12 × 10^−16^	85,567.69	*k* *=* 7.12 × 10^−16^ exp(−10,292/*T*)	0.999

*k*(*T*) = *A* exp(−*E*a/*RT*), where *A*, pre-exponential factor; *E*a, activation energy; *R*, ideal gas constant (*R* = 8.314); *T*, temperature (K); *R*^2^, correlation coefficient.

### 2.3. QSAR Models

**Figure 3:**
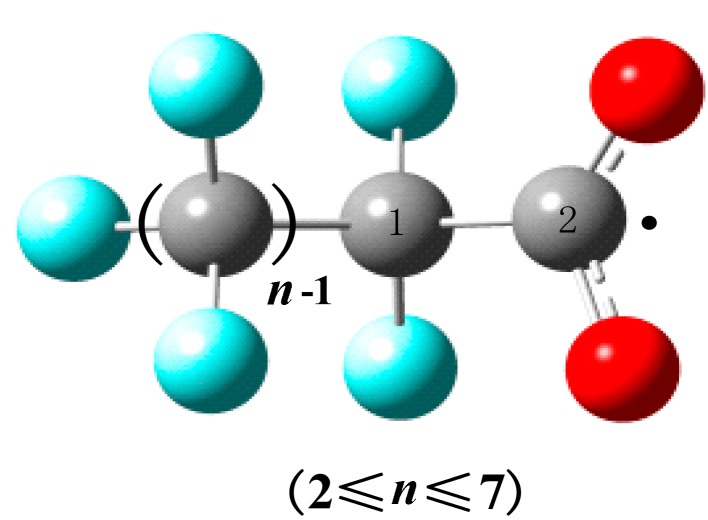
The atom number of PFCAs (2 ≤ *n* ≤ 7) radicals.

The quantitative structure–activity relationship is performed to reveal the relationship between the structures of PFCAs radicals (~C_8_–C_2_) and the rate constants of decarboxylation reactions at 298.15 K. The atom number of PFCAs (2 ≤ *n* ≤ 7) radicals is drawn in Figure 3. The obtained parameters, such as the bond length, molecular mass, volume, dipole, bond energy and net atomic charge, are listed in Table 4. Table 5 shows the actual values, predicted values and residual values of the model. The comparison of actual values and predicted ones is shown in Figure S2.

The independent variables are chosen as follows: the bond length of C1–C2 (*R*_C1–C2_), the molecular mass (*M*), the volume (*V*), the difference Δ*E* between *E*_HOMO_ and *E*_LUMO_, the dipole of molecule, the bond energy of C1–C2 (*E*_C1–C2_), the net atomic charges on atom C_1_ and C_2_ (*Q*_C1_ and *Q*_C2_). The dependent variable is the logarithmic form of rate constants (log *k*). Then, the genetic function approximation (GFA) is adopted to filter the optimum combination of parameters. A five-parameter combination is obtained, which includes sufficient information and high reliability in predicting the rate constants of decarboxylation reactions. The structure–activity model obtained from the GFA calculation is given as follows:

log *k* = 361.39 × *R*_C1–C2_ − 0.03 × *V* − 2.87 × Δ*E* + 0.22 × *E*_C1–C2_ − 27.73 × *Q*_C2_ − 534.97 (1)

The correlative coefficient *R*^2^ and the cross validated *R*^2^ (CV) are above 0.999. It can be seen from Table 5 that the relativity between actual and predicted values is excellent.

As the structure–activity model shows, *R*_C1–C2_ and *E*_C1–C2_ are positively correlated to rate constants, while *V*, Δ*E* and *Q*_C2_ are negatively correlated. It is obvious that the rate constant is largely affected by *R*_C1–C2_ due to the high factor. When the bond of *R*_C1–C2_ is elongated, the stereo effect is reduced, and the rate constant is increased correspondingly.

**Table 4:** The parameters used to make quantitative structure–activity relationship analysis.

PFCAs Radicals	*R*_C1–C2_ (Å)	*M*	*V* (cm^3^/mol)	Δ*E* (a.u.)	Dipole (Debye)	*E*_C1–C2_ (kcal/mol)	*Q*_C1_ (C)	*Q*_C2_ (C)
C_7_F_15_COO	1.5433	412.9659	166.972	0.3634	1.1594	24.12	0.659	0.365
C_6_F_13_COO	1.5386	362.9691	152.634	0.3616	1.1382	24.22	0.663	0.367
C_5_F_11_COO	1.5388	312.9723	141.161	0.2728	1.2436	24.15	0.600	0.374
C_4_F_9_COO	1.5441	262.9755	118.218	0.2533	1.2094	24.10	0.455	0.457
C_3_F_7_COO	1.5394	212.9787	83.114	0.3665	1.2019	24.26	0.472	0.417
C_2_F_5_COO	1.5380	162.9819	62.998	0.2863	1.1352	23.03	0.322	0.418
CF_3_COO	1.5392	112.9850	46.759	0.2894	1.1828	19.69	0.936	0.419

**Table 5:** The actual values, predicted values and residual values of the model (*T* = 298.15 K).

PFCAs Radicals	Lg *k* (Actual Values)	Lg *k* (Predicted Values)	Residual Values
C_7_F_15_COO	12.2430	12.2439	−8.34 × 10^−4^
C_6_F_13_COO	10.9015	10.9029	−1.42 × 10^−3^
C_5_F_11_COO	11.3522	11.3497	2.43 × 10^−3^
C_4_F_9_COO	11.6284	11.6286	−2.02 × 10^−4^
C_3_F_7_COO	11.7627	11.7603	2.43 × 10^−3^
C_2_F_5_COO	11.7340	11.7373	−3.28 × 10^−3^
CF_3_COO	11.8579	11.8571	8.72 × 10^−4^

## 3. Experimental Section

### 3.1. Geometry Optimization

The geometrical parameters of reactants, transition states, intermediates, and products are optimized at the MPWB1K/6-31 + G(d, p) level. The vibrational frequencies have been calculated at the same level in order to determine the nature of stationary points. The MPWB1K method is a hybrid density functional theory (HDFT) model developed by Truhlar *et al.* Study shows that MPWB1K gives the best results for a combination of thermochemistry, thermochemical kinetics, hydrogen bonding and weak interactions, especially for thermochemical kinetics and non-covalent interaction [30]. Compared with other conventional methods such as B3LYP and MP2, MPWB1K is more accurate and less time-consuming. The 6-31+G(d, p) basis sets are chosen to perform the geometry optimization and the 6-311 + G(3df, 2p) basis sets are adopted to calculate the potential energy for this medium-scale system after overall consideration of the computational accuracy and time cost [31,32]. Each transition state is verified to connect the specific reactants with products by performing an intrinsic reaction coordinate (IRC) analysis. All the calculations are performed using the GAUSSIAN 03 programs [33]. In this study, TS, IM and P represent the transition state, the intermediate and product, respectively.

### 3.2. Kinetic Calculation

Among the minimum energy path, about 40 points near the transition state are selected to perform the vibrational frequency calculation, 20 points on the reactant side and 20 points on the product side which should represent the shape of the minimum energy path. Based on the information from *ab initio* calculations, including coordinates, gradients, and force constants or Hessian matrix, the rate constants with tunneling effects are calculated by the POLYRATE 9.7 program [34]. The canonical variational transition state theory (CVT) with small-curvature tunneling (SCT) effect correction is a useful method to calculate rate constants [35], which has been successfully applied to lots of research [36,37,38]. The Rice-Ramsperger-Kassel-Marcus (RRKM) theory is used to calculate the rate constants of the reactions that have no transition states. Study shows that the rate constants of barrierless reactions calculated by RRKM are in good agreement with the experimental observation [39].

### 3.3. QSAR Analysis

The genetic function approximation (GFA) [40] in the Materials studio package is adopted to describe the relationship between the rate constants of decarboxylation reactions and the structures of PFCAs radicals (~C8–C2). The parameters obtained from geometry optimization and frequency calculation, such as the bond length, molecular mass, volume, atomic net charge and frontier orbital energies (*E*_HOMO_, *E*_LUMO_, Δ*E*), are chosen as the independent variables. The dependent variable is the logarithmic form of rate constants, log *k*. The optimal parameter combination can be obtained after analysis.

## 4. Conclusions

In this paper, the rate constants of electrochemical reactions are calculated using RRKM theory and the CVT with SCT correction. The structure–activity relationship is analyzed in order to find out the relationship between the structures of PFCA radicals (~C_2_–C_8_) and the rate constants of decarboxylation reactions.

(1) The circle of C*_n_*F_2*n*+1_ → C*_n_*_−1_F_2*n*−1_ is the optimal reaction pathway in the degradation process due to the high rate constants.

(2) The quantitative structure–activity relationship is investigated using the GFA method. The structure–activity model has been constructed: *R*_C1–C2_ and *E*_C1–C2_ are positively correlated to the rate constants of decarboxylation reactions.

## Supplementary Files

Supplementary File 1

## References

[1] Hori H., Hayakawa E., Einaga H., Kutsuna S., Koike K., Ibusuki T., Kiatagawa H., Arakawa R. (2004). Decomposition of environmentally persistent perfluorooctanoic acid in water by photochemical approaches. Environ. Sci. Technol..

[2] Renner R. (2001). Growing concern over perfluorinated chemicals. Environ. Sci. Technol..

[3] Giesy J.P., Kannan K. (2001). Global distribution of perfluorooctane sulfonate in wildlife. Environ. Sci. Technol..

[4] Schultz M.M., Barofsky D.F., Field J.A. (2003). Fluorinated alkyl surfactants. Environ. Eng. Sci..

[5] DeWitt J.C., Copeland C.B., Strynar M.J., Luebke R.W. (2008). Perfluorooctanoic acid-induced immunomodulation in adult C57BL/6J or C57BL/6N female mice. Environ. Health Perspect..

[6] Lin H., Niu J., Ding S., Zhang L. (2012). Electrochemical degradation of perfluorooctanoic acid (PFOA) by Ti/SnO_2_-Sb, Ti/SnO_2_-Sb/PbO_2_ and Ti/SnO_2_-Sb/MnO_2_ anodes. Water Res..

[7] Loganathan B.G., Sajwan K.S., Sinclair E., Senthil Kumar K., Kannan K. (2007). Perfluoroalkyl sulfonates and perfluorocarboxylates in two wastewater treatment facilities in Kentucky and Georgia. Water Res..

[8] Guo R., Sim W.J., Lee E.S., Lee J.H., Oh J.E. (2010). Evaluation of the fate of perfluoroalkyl compounds in wastewater treatment plants. Water Res..

[9] Loos R., Locoro G., Comero S., Contini S., Schwesig D., Werres F., Balsaa P., Gans O., Weiss S., Blaha L. (2010). Pan-European survey on the occurrence of selected polar organic persistent pollutants in ground water. Water Res..

[10] Falandysz J., Taniyasu S., Gulkowska A., Yamashita N., Schulte-Oehlmann U. (2006). Is fish a major source of fluorinated surfactants and repellents in humans living on the Baltic Coast?. Environ. Sci. Technol..

[11] Keller J.M., Kannan K., Taniyasu S., Yamashita N., Day R.D., Arendt M.D., Segars A.L., Kucklick J.R. (2005). Perfluorinated compounds in the plasma of loggerhead and Kemp’s ridley sea turtles from the southeastern coast of the United States. Environ. Sci. Technol..

[12] Moriwaki H., Takagi Y., Tanaka M., Tsuruho K., Okitsu K., Maeda Y. (2005). Sonochemical decomposition of perfluorooctane sulfonate and perfluorooctanoic acid. Environ. Sci. Technol..

[13] Berthiaume J., Wallace K.B. (2002). Perfluorooctanoate, perflourooctanesulfonate, and *N*-ethyl perfluorooctanesulfonamido ethanol; peroxisome proliferation and mitochondrial biogenesis. Toxicol. Lett..

[14] Olsen G.W., Logan P.W., Hansen K.J., Simpson C.A., Burris J.M., Burlew M.M., Vorarath P.P., Venkateswarlu P., Schumpert J.C., Mandel J.H. (2003). An occupational exposure assessment of a perfluorooctanesulfonyl fluoride production site: Biomonitoring. AIHA J..

[15] Sanderson H., Boudreau T.M., Mabury S.A., Cheong W.J., Solomon K.R. (2002). Ecological impact and environmental fate of perfluorooctane sulfonate on the zooplankton community in indoor microcosms. Environ. Toxicol. Chem..

[16] Hines E.P., White S.S., Stanko J.P., Gibbs-Flournoy E.A., Lau C., Fenton S.E. (2009). Phenotypic dichotomy following developmental exposure to perfluorooctanoic acid (PFOA) in female CD-1 mice: Low doses induce elevated serum leptin and insulin, and overweight in mid-life. Mol. Cell. Endocrinol..

[17] Kennedy G.L., Butenhoff J.L., Olsen G.W., O’Connor J.C., Seacat A.M., Perkins R.G., Biegel L.B., Murphy S.R., Farrar D.G. (2004). The toxicology of perfluorooctanoate. Crit. Rev. Toxicol..

[18] Lau C., Anitole K., Hodes C., Lai D., Pfahles-Hutchens A., Seed J. (2007). Perfluoroalkyl acids: A review of monitoring and toxicological findings. J. Toxicol. Sci..

[19] US EPA Revised Draft Hazard Assessment of Perfluorooctanoic Acid and Its Salts. http://www.fluoridealert.org/wp-content/pesticides/pfoa.epa.nov.4.2002.pdf.

[20] Järnberg U., Holmström K., van Bavel B. Perfluoroalkylated Acids and Related Compounds (PFAS) in the Swedish Environment-Chemistry. http://www.diva-portal.org/smash/get/diva2:657980/FULLTEXT01.pdf.

[21] Hoffman K., Webster T.F., Bartell S.M., Weisskopf M.G., Fletcher T., Vieira V.M. (2011). Private drinking water wells as a source of exposure to perfluorooctanoic acid (PFOA) in communities surrounding a fluoropolymer production facility. Environ. Health Perspect..

[22] Ochiai T., Moriyama H., Nakata K., Murakami T., Koide Y., Fujishima A. (2011). Electrochemical and photocatalytic decomposition of perfluorooctanoic acid with a hybrid reactor using a boron-doped diamond electrode and TiO_2_ photocatalyst. Chem. Lett..

[23] Zhuo Q., Deng S., Yang B., Huang J., Yu G. (2011). Efficient electrochemical oxidation of perfluorooctanoate using a Ti/SnO_2_-Sb-Bi anode. Environ. Sci. Technol..

[24] Ochiai T., Lizuka Y., Nakata K., Murakami T., Tryk D.A., Fujishima A., Koide Y., Morito Y. (2011). Efficient electrochemical decomposition of perfluorocarboxylic acids by the use of a boron-doped diamond electrode. Diam. Relat. Mater..

[25] Niu J., Lin H., Gong C., Sun X. (2013). Theoretical and experimental insights into the electrochemical mineralization mechanism of perfluorooctanoic acid. Environ. Sci. Technol..

[26] Niu J., Lin H., Xu J., Wu H., Li Y. (2012). Electrochemical mineralization of perfluorocarboxylic acids (PFCAs) by Ce-doped modified porous nanocrystalline PbO_2_ film electrode. Environ. Sci. Technol..

[27] Zhang Q., Zhang R., Gu Y. (2004). Kinetics and mechanism of O (^3^P) reaction with CH3CHF2: A theoretical study. J. Phys. Chem. A.

[28] Zhang Q., Zhang R., Chan K., Bello I. (2005). Ab initio and variational transition state approach to β-C_3_N_4_ formation: Kinetics for the reaction of CH_3_NH_2_ with H. J. Phys. Chem. A.

[29] Sun T., Zhang Q., Qu X., Wang W. (2005). Mechanism and direct dynamics studies for the reaction of monoethylsilane EtSiH3 with atomic O (^3^P). Chem. Phys. Lett..

[30] Zhao Y., Truhlar D.G. (2004). Hybrid meta density functional theory methods for thermochemistry, thermochemical kinetics, and noncovalent interactions: The MPW1B95 and MPWB1K models and comparative assessments for hydrogen bonding and van der Waals interactions. J. Phys. Chem. A.

[31] Hehre W., Ditchfield R., Pople J. (1972). Theoretical investigations on the solvation process. J. Chem. Phys..

[32] Zheng J.J., Zhao Y., Truhlar D.G. (2009). The DBH24/08 database and its use to assess electronic structure model chemistries for chemical reaction barrier heights. J. Chem. Theory Comput..

[33] Frisch M., Trucks G., Schlegel H., Scuseria G., Robb M., Cheeseman J., Zakrzewski V., Montgomery J., Stratmann R., Burant J. Gaussian 03,Revision E.01. http://www.gaussian.com/g_misc/g03/citation_g03.htm.

[34] Corchado J.C., Chuang Y.Y., Fast P.L., Hu W.P., Liu Y.P., Lynch G.C., Nguyen K.A., Jackels C.F., Fernandez Ramos A., Ellingson B.A. (2007). POLYRATE Version 9.7.

[35] Miller W.H. (1979). Tunneling corrections to unimolecular rate constants, with application to formaldehyde. J. Am. Chem. Soc..

[36] Zhang Q., Yu W., Zhang R., Zhou Q., Gao R., Wang W. (2010). Quantum chemical and kinetic study on dioxin formation from the 2, 4, 6-TCP and 2, 4-DCP precursors. Environ. Sci. Technol..

[37] Qu X., Wang H., Zhang Q., Shi X., Xu F., Wang W. (2009). Mechanistic and kinetic studies on the homogeneous gas-phase formation of PCDD/Fs from 2, 4, 5-trichlorophenol. Environ. Sci. Technol..

[38] Gong C., Sun X., Zhang C. (2013). The atmospheric chemical reaction of 4-*tert*-butylphenol initiated by OH radicals. Environ. Chem..

[39] Hou H., Wang B. (2007). *Ab initio* study of the reaction of propionyl (C_2_H_5_CO) radical with oxygen (O_2_). J. Chem. Phys..

[40] Rogers D., Hopfinger A.J. (1994). Application of genetic function approximation to quantitative structure-activity relationships and quantitative structure-property relationships. J. Chem. Inf. Comput. Sci..

