# Supplementary material for: Kinetics and Quantitative Structure—Activity Relationship Study on the Degradation Reaction from Perfluorooctanoic Acid to Trifluoroacetic Acid
Source: Int J Mol Sci. 2014 Aug 14;15(8):14153–65. doi: 10.3390/ijms150814153 (PMC4159843; doi:10.3390/ijms150814153)
Supplement: Supplementary File 1 [file ijms-15-14153-s001.pdf]

Supplementary Information

Figure S1. The chemical structures of the transition states in electrochemical degradation reactions with the bond lengths (Å).

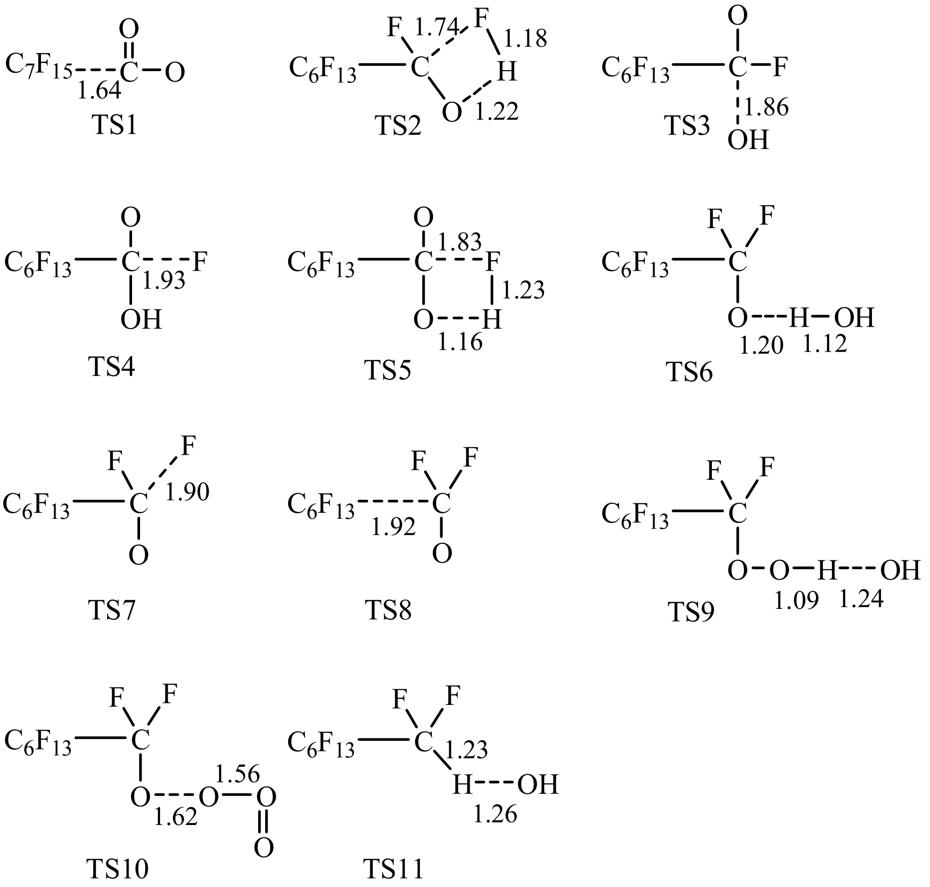

Figure S2. The comparison of actual values and predicted values (T = 298.15 K).

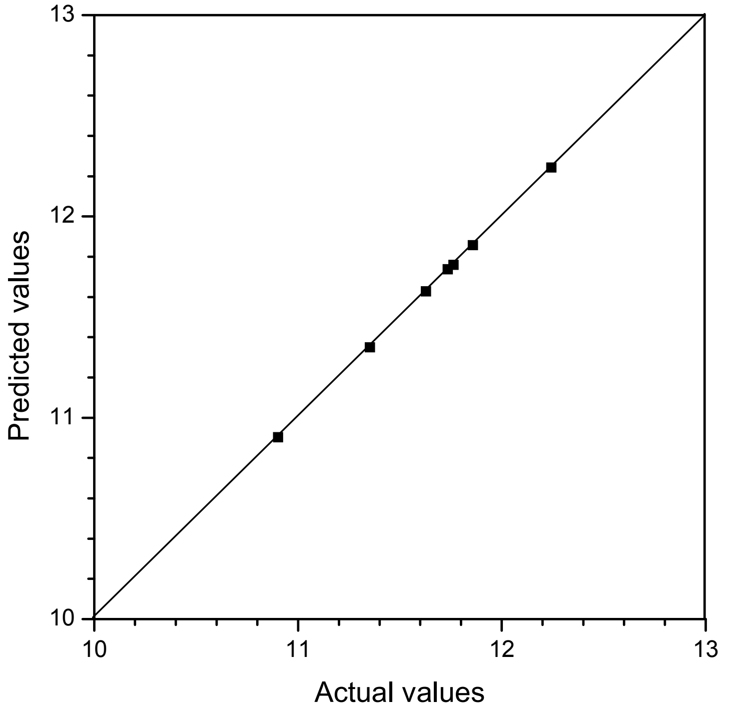

Table S1. The TST, CVT with the ZCT or the SCT correction rate constants of reaction (2).

T (K)	k_{TST} [a]	k_{CVT} [a]	$k_{CVT/ZCT}$ [a]	$k_{CVT/SCT}$ [a]
200	3.10×10^{14}	1.09×10^{12}	1.13×10^{12}	1.13×10^{12}
220	2.65×10^{14}	1.23×10^{12}	1.26×10^{12}	1.26×10^{12}
240	2.34×10^{14}	1.36×10^{12}	1.39×10^{12}	1.39×10^{12}
260	2.12×10^{14}	1.49×10^{12}	1.52×10^{12}	1.52×10^{12}
280	1.95×10^{14}	1.62×10^{12}	1.64×10^{12}	1.64×10^{12}
298.15	1.83×10^{14}	1.72×10^{12}	1.75×10^{12}	1.75×10^{12}
320	1.72×10^{14}	1.85×10^{12}	1.87×10^{12}	1.87×10^{12}
340	1.64×10^{14}	1.95×10^{12}	1.97×10^{12}	1.97×10^{12}
360	1.57×10^{14}	2.06×10^{12}	2.08×10^{12}	2.08×10^{12}
380	1.52×10^{14}	2.15×10^{12}	2.17×10^{12}	2.17×10^{12}
400	1.47×10^{14}	2.25×10^{12}	2.27×10^{12}	2.27×10^{12}
450	1.38×10^{14}	2.47×10^{12}	2.48×10^{12}	2.48×10^{12}
500	1.33×10^{14}	2.66×10^{12}	2.68×10^{12}	2.68×10^{12}

[a] The unit of the rate constant is s^{-1}.

Table S2. The TST, CVT with the ZCT or the SCT correction rate constants of reaction (4).

T (K)	k_{TST} [a]	k_{CVT} [a]	$k_{CVT/ZCT}$ [a]	$k_{CVT/SCT}$ [a]
200	1.78×10^{-39}	7.14×10^{-42}	2.80×10^{-42}	2.80×10^{-42}
220	9.18×10^{-35}	4.69×10^{-37}	1.98×10^{-37}	1.98×10^{-37}
240	7.81×10^{-31}	4.84×10^{-33}	2.18×10^{-33}	2.18×10^{-33}
260	1.66×10^{-27}	1.20×10^{-29}	5.74×10^{-30}	5.74×10^{-30}
280	1.18×10^{-24}	9.78×10^{-27}	4.90×10^{-27}	4.90×10^{-27}
298.15	2.14×10^{-22}	1.96×10^{-24}	1.02×10^{-24}	1.02×10^{-24}
320	5.15×10^{-20}	5.23×10^{-22}	2.84×10^{-22}	2.84×10^{-22}
340	4.20×10^{-18}	1.45×10^{-19}	5.84×10^{-20}	5.84×10^{-20}
360	2.11×10^{-16}	7.67×10^{-18}	3.25×10^{-18}	3.25×10^{-18}
380	7.00×10^{-15}	2.67×10^{-16}	1.18×10^{-16}	1.18×10^{-16}
400	1.64×10^{-13}	6.53×10^{-15}	3.01×10^{-15}	3.01×10^{-15}
450	1.29×10^{-10}	5.55×10^{-12}	2.78×10^{-12}	2.79×10^{-12}
500	2.68×10^{-8}	1.22×10^{-9}	6.58×10^{-10}	6.58×10^{-10}

[a] The unit of the rate constant is s^{-1}.

Table S3. The TST, CVT with the ZCT or the SCT correction rate constants of reaction (5).

T (K)	k_{TST} [b]	k_{CVT} [b]	$k_{CVT/ZCT}$ [b]	$k_{CVT/SCT}$ [b]
200	1.08×10^{-21}	1.48×10^{-22}	1.06×10^{-22}	1.06×10^{-22}
220	3.74×10^{-21}	5.06×10^{-22}	3.71×10^{-22}	3.71×10^{-22}
240	1.06×10^{-20}	1.42×10^{-21}	1.06×10^{-21}	1.06×10^{-21}
260	2.59×10^{-20}	3.41×10^{-21}	2.59×10^{-21}	2.59×10^{-21}
280	5.59×10^{-20}	7.28×10^{-21}	5.62×10^{-21}	5.62×10^{-21}
298.15	1.03×10^{-19}	1.33×10^{-20}	1.04×10^{-20}	1.04×10^{-20}
320	1.99×10^{-19}	2.54×10^{-20}	2.01×10^{-20}	2.01×10^{-20}
340	3.39×10^{-19}	4.29×10^{-20}	3.44×10^{-20}	3.44×10^{-20}
360	5.47×10^{-19}	6.86×10^{-20}	5.56×10^{-20}	5.56×10^{-20}

Table S3. *Cont.*

T (K)	k_{TST} [b]	k_{CVT} [b]	$k_{CVT/ZCT}$ [b]	$k_{CVT/SCT}$ [b]
380	8.43×10^{-19}	1.05×10^{-19}	8.59×10^{-20}	8.60×10^{-20}
400	1.25×10^{-18}	1.53×10^{-19}	1.05×10^{-19}	1.06×10^{-19}
450	2.93×10^{-18}	3.45×10^{-19}	2.44×10^{-19}	2.44×10^{-19}
500	5.92×10^{-18}	6.75×10^{-19}	4.89×10^{-19}	4.89×10^{-19}

[b] The unit of the rate constant is $cm^3 molecule^{-1} s^{-1}$.

Table S4. The TST, CVT with the ZCT or the SCT correction rate constants of reaction (6).

T (K)	k_{TST} [a]	k_{CVT} [a]	$k_{CVT/ZCT}$ [a]	$k_{CVT/SCT}$ [a]
200	1.14×10^{-19}	1.83×10^{-20}	1.64×10^{-20}	1.64×10^{-20}
220	7.42×10^{-17}	1.20×10^{-17}	4.83×10^{-18}	4.83×10^{-18}
240	1.65×10^{-14}	2.59×10^{-15}	1.12×10^{-15}	1.12×10^{-15}
260	1.60×10^{-12}	2.46×10^{-13}	1.13×10^{-13}	1.13×10^{-13}
280	8.06×10^{-11}	1.22×10^{-11}	5.89×10^{-12}	5.89×10^{-12}
298.15	1.80×10^{-9}	2.69×10^{-10}	1.35×10^{-10}	1.35×10^{-10}
320	4.74×10^{-8}	6.97×10^{-9}	3.63×10^{-9}	3.63×10^{-9}
340	6.56×10^{-7}	9.52×10^{-8}	5.12×10^{-8}	5.12×10^{-8}
360	6.79×10^{-6}	9.72×10^{-7}	5.37×10^{-7}	5.37×10^{-7}
380	5.49×10^{-5}	7.78×10^{-6}	4.40×10^{-6}	4.40×10^{-6}
400	3.61×10^{-4}	5.06×10^{-5}	2.93×10^{-5}	2.93×10^{-5}
450	1.92×10^{-2}	2.63×10^{-3}	1.59×10^{-3}	1.59×10^{-3}
500	4.63×10^{-1}	6.20×10^{-2}	3.87×10^{-2}	3.87×10^{-2}

[a] The unit of the rate constant is s^{-1}.

Table S5. The TST, CVT with the ZCT or the SCT correction rate constants of reaction (7).

T (K)	k_{TST} [a]	k_{CVT} [a]	$k_{CVT/ZCT}$ [a]	$k_{CVT/SCT}$ [a]
200	1.16×10^{-29}	1.44×10^{-32}	1.54×10^{-32}	1.54×10^{-32}
220	7.08×10^{-26}	1.12×10^{-28}	1.18×10^{-28}	1.18×10^{-28}
240	1.02×10^{-22}	1.96×10^{-25}	2.04×10^{-25}	2.04×10^{-25}
260	4.81×10^{-20}	1.08×10^{-22}	1.12×10^{-22}	1.12×10^{-22}
280	9.46×10^{-18}	2.42×10^{-20}	2.49×10^{-20}	2.49×10^{-20}
298.15	6.19×10^{-16}	1.75×10^{-18}	1.79×10^{-18}	1.79×10^{-18}
320	5.08×10^{-14}	1.59×10^{-16}	1.62×10^{-16}	1.62×10^{-16}
340	1.75×10^{-12}	5.91×10^{-15}	6.01×10^{-15}	6.01×10^{-15}
360	4.08×10^{-11}	1.47×10^{-13}	1.49×10^{-13}	1.49×10^{-13}
380	6.85×10^{-10}	2.61×10^{-12}	2.64×10^{-12}	2.64×10^{-12}
400	8.67×10^{-9}	3.46×10^{-11}	3.50×10^{-11}	3.50×10^{-11}
450	1.85×10^{-6}	8.14×10^{-9}	8.21×10^{-9}	8.21×10^{-9}
500	1.36×10^{-4}	6.41×10^{-7}	6.45×10^{-7}	6.45×10^{-7}

[a] The unit of the rate constant is s^{-1}.

Table S6. The TST, CVT with the ZCT or the SCT correction rate constants of reaction (8).

T (K)	k_{TST} [b]	k_{CVT} [b]	$k_{CVT/ZCT}$ [b]	$k_{CVT/SCT}$ [b]
200	5.11×10^{-22}	3.42×10^{-25}	1.10×10^{-24}	1.15×10^{-24}
220	2.86×10^{-21}	2.41×10^{-24}	6.47×10^{-24}	6.70×10^{-24}
240	1.21×10^{-20}	1.24×10^{-23}	2.87×10^{-23}	2.96×10^{-23}
260	4.15×10^{-20}	4.95×10^{-23}	1.02×10^{-22}	1.05×10^{-22}
280	1.20×10^{-19}	1.63×10^{-22}	3.06×10^{-22}	3.14×10^{-22}
298.15	2.80×10^{-19}	4.20×10^{-22}	7.36×10^{-22}	7.52×10^{-22}
320	6.89×10^{-19}	1.15×10^{-21}	1.87×10^{-21}	1.91×10^{-21}
340	1.43×10^{-18}	2.57×10^{-21}	3.98×10^{-21}	4.05×10^{-21}
360	2.75×10^{-18}	5.30×10^{-21}	7.83×10^{-21}	7.95×10^{-21}
380	4.95×10^{-18}	1.02×10^{-20}	1.44×10^{-20}	1.46×10^{-20}
400	8.46×10^{-18}	1.83×10^{-20}	2.52×10^{-20}	2.55×10^{-20}
450	2.67×10^{-17}	6.44×10^{-20}	8.30×10^{-20}	8.39×10^{-20}
500	6.87×10^{-17}	1.80×10^{-19}	2.21×10^{-19}	2.23×10^{-19}

[b] The unit of the rate constant is $cm^3 \cdot molecule^{-1} \cdot s^{-1}$.

Table S7. The TST, CVT with the ZCT or the SCT correction rate constants of reaction (9).

T (K)	k_{TST} [a]	k_{CVT} [a]	$k_{CVT/ZCT}$ [a]	$k_{CVT/SCT}$ [a]
200	5.30×10^{-23}	6.53×10^{-24}	4.21×10^{-24}	4.21×10^{-24}
220	9.65×10^{-20}	1.14×10^{-20}	7.50×10^{-21}	7.50×10^{-21}
240	5.09×10^{-17}	5.76×10^{-18}	3.86×10^{-18}	3.86×10^{-18}
260	1.03×10^{-14}	1.12×10^{-15}	7.59×10^{-16}	7.59×10^{-16}
280	9.84×10^{-13}	1.04×10^{-13}	7.02×10^{-14}	7.02×10^{-14}
298.15	3.65×10^{-11}	3.74×10^{-12}	2.52×10^{-12}	2.52×10^{-12}
320	1.65×10^{-9}	1.63×10^{-10}	1.09×10^{-10}	1.09×10^{-10}
340	3.52×10^{-8}	3.39×10^{-9}	2.24×10^{-9}	2.24×10^{-9}
360	5.37×10^{-7}	5.03×10^{-8}	3.29×10^{-8}	3.29×10^{-8}
380	6.17×10^{-6}	5.61×10^{-7}	3.62×10^{-7}	3.62×10^{-7}
400	5.55×10^{-5}	4.92×10^{-6}	3.14×10^{-6}	3.14×10^{-6}
450	5.79×10^{-3}	4.78×10^{-4}	2.95×10^{-4}	2.95×10^{-4}
500	2.40×10^{-1}	1.86×10^{-2}	1.15×10^{-2}	1.15×10^{-2}

[a] The unit of the rate constant is s^{-1}.

Table S8. The TST, CVT with the ZCT or the SCT correction rate constants of reaction (10).

T (K)	k_{TST} [a]	k_{CVT} [a]	$k_{CVT/ZCT}$ [a]	$k_{CVT/SCT}$ [a]
200	6.58×10^{7}	9.80×10^{6}	9.70×10^{6}	9.70×10^{6}
220	2.00×10^{8}	3.02×10^{7}	2.99×10^{7}	2.99×10^{7}
240	5.07×10^{8}	7.78×10^{7}	7.70×10^{7}	7.70×10^{7}
260	1.12×10^{9}	1.74×10^{8}	1.72×10^{8}	1.72×10^{8}
280	2.23×10^{9}	3.49×10^{8}	3.46×10^{8}	3.46×10^{8}
298.15	3.84×10^{9}	6.07×10^{8}	6.01×10^{8}	6.01×10^{8}
320	6.86×10^{9}	1.09×10^{9}	1.08×109	1.08×10^{9}
340	1.09×10^{10}	1.75×10^{9}	1.73×10^{9}	1.73×10^{9}

Table S8. *Cont.*

T (K)	k_{TST} [a]	k_{CVT} [a]	$k_{CVT/ZCT}$ [a]	$k_{CVT/SCT}$ [a]
360	1.66×10^{10}	2.67×10^{9}	2.65×10^{9}	2.65×10^{9}
380	2.41×10^{10}	3.90×10^{9}	3.87×10^{9}	3.87×10^{9}
400	3.39×10^{10}	5.50×10^{9}	5.45×10^{9}	5.45×10^{9}
450	6.97×10^{10}	1.14×10^{10}	1.13×10^{10}	1.13×10^{10}
500	1.25×10^{11}	2.05×10^{10}	2.03×10^{10}	2.03×10^{10}

[a] The unit of the rate constant is s^{-1}.

Table S9. The TST, CVT with the ZCT or the SCT correction rate constants of reaction (12).

T (K)	k_{TST} [b]	k_{CVT} [b]	$k_{CVT/ZCT}$ [b]	$k_{CVT/SCT}$ [b]
200	3.16×10^{-45}	1.65×10^{-62}	1.14×10^{-59}	1.21×10^{-58}
220	1.54×10^{-42}	1.25×10^{-59}	4.16×10^{-57}	3.50×10^{-56}
240	2.69×10^{-40}	3.05×10^{-57}	5.59×10^{-55}	3.85×10^{-54}
260	2.14×10^{-38}	3.12×10^{-55}	3.48×10^{-53}	2.03×10^{-52}
280	9.20×10^{-37}	1.62×10^{-53}	1.19×10^{-51}	6.00×10^{-51}
298.15	1.82×10^{-35}	3.64×10^{-52}	1.92×10^{-50}	8.70×10^{-50}
320	4.25×10^{-34}	9.57×10^{-51}	3.58×10^{-49}	1.45×10^{-48}
340	5.37×10^{-33}	1.31×10^{-49}	3.73×10^{-48}	1.37×10^{-47}
360	5.16×10^{-32}	1.33×10^{-48}	2.98×10^{-47}	1.01×10^{-46}
380	3.93×10^{-31}	1.05×10^{-47}	1.91×10^{-46}	6.05×10^{-46}
400	2.46×10^{-30}	6.78×10^{-47}	1.02×10^{-45}	3.01×10^{-45}
450	1.21×10^{-28}	3.41×10^{-45}	3.44×10^{-44}	8.93×10^{-44}
500	2.80×10^{-27}	7.83×10^{-44}	5.76×10^{-43}	1.35×10^{-42}

[b] The unit of the rate constant is $cm^3 \cdot molecule^{-1} \cdot s^{-1}$.

Table S10. The TST, CVT with the ZCT or the SCT correction rate constants of reaction (14).

T (K)	k_{TST} [b]	k_{CVT} [b]	$k_{CVT/ZCT}$ [b]	$k_{CVT/SCT}$ [b]
200	3.69×10^{-69}	1.39×10^{-70}	2.60×10^{-70}	2.60×10^{-70}
220	2.63×10^{-64}	1.07×10^{-65}	1.80×10^{-65}	1.80×10^{-65}
240	2.95×10^{-60}	1.28×10^{-61}	1.98×10^{-61}	1.98×10^{-61}
260	7.95×10^{-57}	3.63×10^{-58}	5.26×10^{-58}	5.27×10^{-58}
280	7.01×10^{-54}	3.33×10^{-55}	4.59×10^{-55}	4.59×10^{-55}
298.15	1.51×10^{-51}	7.41×10^{-53}	9.84×10^{-53}	9.84×10^{-53}
320	4.39×10^{-49}	2.22×10^{-50}	2.84×10^{-50}	2.84×10^{-50}
340	4.20×10^{-47}	2.17×10^{-48}	2.70×10^{-48}	2.70×10^{-48}
360	2.44×10^{-45}	1.28×10^{-46}	1.56×10^{-46}	1.56×10^{-46}
380	9.28×10^{-44}	4.98×10^{-45}	5.93×10^{-45}	5.93×10^{-45}
400	2.47×10^{-42}	1.35×10^{-43}	1.57×10^{-43}	1.57×10^{-43}
450	2.59×10^{-39}	1.45×10^{-40}	1.64×10^{-40}	1.64×10^{-40}
500	6.93×10^{-37}	3.96×10^{-38}	4.37×10^{-38}	4.37×10^{-38}

[b] The unit of the rate constant is $cm^3 \cdot molecule^{-1} \cdot s^{-1}$.

Table S11. The TST, CVT with the ZCT or the SCT correction rate constants of reaction (15).

T (K)	k_{TST} [b]	k_{CVT} [b]	$k_{CVT/ZCT}$ [b]	$k_{CVT/SCT}$ [b]
200	4.84×10^{-36}	6.06×10^{-37}	3.38×10^{-38}	3.73×10^{-38}
220	3.97×10^{-34}	4.88×10^{-35}	3.32×10^{-36}	3.63×10^{-36}
240	1.58×10^{-32}	1.91×10^{-33}	1.54×10^{-34}	1.67×10^{-34}
260	3.60×10^{-31}	4.31×10^{-32}	4.04×10^{-33}	4.33×10^{-33}
280	5.31×10^{-30}	6.27×10^{-31}	6.70×10^{-32}	7.15×10^{-32}
298.15	4.50×10^{-29}	5.26×10^{-30}	6.26×10^{-31}	6.64×10^{-31}
320	4.30×10^{-28}	4.98×10^{-29}	6.66×10^{-30}	7.02×10^{-30}
340	2.66×10^{-27}	3.05×10^{-28}	4.49×10^{-29}	4.71×10^{-29}
360	1.35×10^{-26}	1.54×10^{-27}	2.47×10^{-28}	2.58×10^{-28}
380	5.82×10^{-26}	6.60×10^{-27}	1.15×10^{-27}	1.19×10^{-27}
400	2.18×10^{-25}	2.46×10^{-26}	4.59×10^{-27}	4.77×10^{-27}
450	3.63×10^{-24}	4.04×10^{-25}	8.84×10^{-26}	9.11×10^{-26}
500	3.55×10^{-23}	3.91×10^{-24}	9.75×10^{-25}	1.00×10^{-24}

[b] The unit of the rate constant is $cm^3 \cdot molecule^{-1} \cdot s^{-1}$.
